# Large Unbalanced Credit Scoring Using Lasso-Logistic Regression Ensemble

**DOI:** 10.1371/journal.pone.0117844

**Published:** 2015-02-23

**Authors:** Hong Wang, Qingsong Xu, Lifeng Zhou

**Affiliations:** School of Mathematics & Statistics, Central South University, Changsha, Hunan, China; Queen’s University Belfast, UNITED KINGDOM

## Abstract

Recently, various ensemble learning methods with different base classifiers have been proposed for credit scoring problems. However, for various reasons, there has been little research using logistic regression as the base classifier. In this paper, given large unbalanced data, we consider the plausibility of ensemble learning using regularized logistic regression as the base classifier to deal with credit scoring problems. In this research, the data is first balanced and diversified by clustering and bagging algorithms. Then we apply a Lasso-logistic regression learning ensemble to evaluate the credit risks. We show that the proposed algorithm outperforms popular credit scoring models such as decision tree, Lasso-logistic regression and random forests in terms of AUC and F-measure. We also provide two importance measures for the proposed model to identify important variables in the data.

## Introduction

Credit scoring analyzes the characteristics and performance of past loans and predicts the delinquency probability of loan applicants in the near future based on age, financial flows and repayment records and other characteristics [1]. The ability to discriminate good customers from bad ones is of extreme importance for lending companies or organizations and an improvement in prediction accuracy of even a percent may indicate a huge gain in profit [2].

The pioneer work to discriminate between good and bad loans using statistical method was due to [3]. Since then and particularly after the invention of credit cards in the late 1960s, various state-of-the-art machine learning and sophisticated statistical methods have been proposed to tackle the classification issue in application scoring. Among them, the most popular ones are linear discriminant analysis [4], logistic regression [5], neural networks [6, 7], decision trees [8], and support vector machines [9]. Comprehensive reviews of these methods can be found in [10–14].

Due to the success of ensemble methods in various classification tasks [15–17], ensemble algorithms have also found a place in credit scoring modeling and experimental results have revealed that ensemble methods can substantially improve the performance [18–21]. One of the first works on credit scoring using ensemble algorithms is due to [22]. In their work, an ensemble of overlapping classification trees was introduced and a Markov Chain Monte Carlo procedure was developed to choose different tree structures. Later on, a neural network ensemble algorithm using negative correlation was proposed in [23]. However, in terms of average prediction accuracy [24], multiple neural networks classifiers may not outperform a single best neural networks classifier in many cases. Beside neural networks, support vector machines (SVM) are frequently chosen as the base learners in credit scoring ensemble algorithms [18, 25]. As is common to all SVM methods, SVM has a difficulty in illustrating the variables’ importance, and suffers from the overfitting problem if its parameters are not properly chosen. In addition, it needs much time and efforts to construct a best hyper-plane, particularly when facing large datasets. Logistic regression, one of the most popular statistical methods in credit scoring [26], is also used as a benchmark base learner in some aforementioned studies. However, only classical logistic regression models have been applied so far in application credit scoring.

A very common situation in real-life credit scoring applications is that the data collected are usually highly unbalanced or skewed, i.e. most examples in the data belong to one class (the majority or the negative class) and the rest belong to the other class (the minority or the positive class). Standard learning algorithms are in favor of the majority class, and they will usually show poor performance on the minority class examples. An ideal credit scoring model should perform equally well both on the minority class and majority class. Compared to a large number of studies in credit scoring, there is relatively only a little research focusing on scoring imbalanced data. Cost-sensitive learning and re-sampling approaches are two general methods applied to alleviate the class imbalance problem. Cost-sensitive learning algorithms which incorporate costs into certain classifiers usually assume that the misclassification costs are known beforehand and require special knowledge of the classifiers themselves [27]. However, these two conditions are not easily satisfied in practice. Hence, most credit scoring algorithms have adopted a re-sampling approach [21, 28–32]. In [21, 28], simple over-sampling or under-sampling approaches are applied and the experiments in [29, 30] provide evidence that over-sampling is superior to under-sampling in terms of accuracy. The results in [31] also reveal that over-sampling outperforms under-sampling in most cases, especially with the logistic regression model. In [32], a particular version of bagging called subagging which is suitable for imbalanced learning is applied, however, a parameter controlling the proportion of positive and negative examples needs to be specified beforehand. Due to the complexity of the problem, imbalanced data classification is not fully solved in aforementioned studies and are still in need of further refinement and research.

On the other hand, big data containing millions or even billions of customers application records have become a commonplace for major credit institutions. If all the data are used to train the classification model, it would be very inefficient and time consuming. How to exploit efficiently the huge amount of data to train a satisfactory model within reasonable time and common computing facilities has become more and more imperative for banks and other credit companies.

In this paper, to demonstrate the effeteness of ensemble learning and Lasso-logistic regression(LLR) in tackling the large unbalanced data classification problem in credit scoring, a Lasso-logistic regression ensemble(LLRE) learning algorithm is proposed. First, majority class examples in the dataset are clustered into a number of subgroups, then a number of balanced and “smaller” training sets are formed by each majority subgroup and a bagged version of the whole minority data. Next, base learners namely, Lasso-logistic regression models are trained with these smaller training sets. Different from traditional cross-validation methods [33], the *λ* parameter in Lasso algorithm is determined by the current so-called OBS data. The OBS data are then used to evaluate the base classifiers’ performance. Finally, the Lasso-logistic models are combined together into the ensemble using a weighted average scheme. As the AUC(Area Under the Curve) metric is independent of class distribution and is more suitable than accuracy in the scenario of unbalanced learning [9, 34], we choose AUC as the evaluation metric in our experiment. F-measure, a tradeoff between precision and recall, is also chosen. Results show that the proposed algorithm outperforms decision tree, Lasso-logistic regression and popular ensemble learning algorithms such as random forests in terms of both AUC and F-measure.

Besides detecting the default customers among all loan applicants, financial institutions also have strong interest in determining which characteristics of the applicants are the most significant ones that affecting the default probability. Thus, as a minor contribution, we provide two kind of measures in LLRE for evaluating the variable importance. The first so-called LLR-occurrence measure is based on the variable selection feature of the Lasso algorithm. The second mean AUC decrease measure, inspired by [35], depends on AUC values before and after variable permutations. We apply these two measures for evaluating the top ranking variables that are important for credit scoring.

## Background

In this section, we first give a brief introduction of Lasso-logistic regression in the credit scoring scenario and then we discuss a particular ensemble learning-the bagging algorithm. Later on, we shall develop our credit scoring ensemble algorithm using both Lasso-logistic regression and bagging.

### Lasso-logistic regression

Application credit scoring determines the probability that a credit applicant will default on his/her credit obligation. From a statistical learning and data mining point of view, application credit scoring can be viewed as a binary classification problem. Suppose that we have credit scoring data (**x** ^*i*^, *y*_*i*_), *i* ∈ 1, 2, ⋯, *n*, where **x** ^*i*^ = (*x*_*i*1_, *x*_*i*2_, ⋯, *x*_*ip*_) are predictor variables of applicants and *y*_*i*_ are class labels(binary responses,1 for default and 0 for non-default). We want to determine the conditional probability *p*(y∣x) of a specific applicant belonging to a class(0 or 1), given the variables of that credit applicant. Rather than modeling the response y directly, logistic regression computes the probability that y belongs to a particular class label via a linear function of features x:

$$\begin{array}{c} \log\frac{\mathrm{Prob}(Y=1|x)}{\mathrm{Prob}(Y=0|x)}=\beta_{0}+x^{T}\beta \end{array}$$ (1)

where *β*_0_ denotes the intercept, *β* = (*β*_1_, ⋯, *β*_*p*_) denotes the linear coefficients, and Prob(*Y* = 1∣*x*), Prob(*Y* = 0∣*x*) denote the conditional probabilities of the class labels 1, 0 respectively.

A maximum likelihood approach is commonly used in calculating the coefficients and the log-likelihood can be written:

$$\begin{array}{ccc} l(\beta_{0},\beta ) & = & \sum_{i=1}^{n}\{y_{i}logProb\left(Y=1;\beta \right)\\ & & +\left(1-y_{i}\right)log\left(1-Prob\left(Y=1;\beta \right)\right)\}\\ & = & \sum_{i=1}^{n}\{y_{i}\left(\beta_{0}+x_{i}^{T}\beta \right)\\ & & -log(1+e^{\beta_{0}+x_{i}^{T}\beta )}\} \end{array}$$ (2)

The above logistic regression model can be further extended into Lasso logistic regression model by imposing an *L*_1_ constraint on *β* parameters [33, 36] and the problem is to minimize the negative log-likelihood function with the penalty term

$$\begin{array}{c} \sum_{i=1}^{n}\left\{log(1+e^{\beta_{0}+x_{i}^{T}\beta )}-y_{i}\left(\beta_{0}+x_{i}^{T}\beta \right)\right\}+\lambda \sum_{j=1}^{p}|\beta_{j}| \end{array}$$ (3)

Due to the above constraint, making *λ* sufficiently large will cause some coefficients in *β* become zero. Depending on the value of *λ*, a Lasso model can include any number of variables. In this way, both shrinkage and feature (variable) selection are done simultaneously and it is also this property that makes Lasso generally much easy to interpret and a very popular algorithm.

To date, there is no reported research using the latest regularized logistic regression models such as Lasso as the base learning algorithm, tough Lasso itself is efficient in dealing with large datasets and convenient for screening variables [33].

### Ensemble learning

Ensemble learning is a kind of machine learning paradigm in which multiple models, such as decision trees, neural networks and SVM, are combined together to solve a particular problem [37]. Typical ensemble methods including AdaBoost [38], bagging [39], random forests [40] and gradient boosted machines [41]. And all these methods encourage diversity of the base learners to some extent to compensate individual errors and reach a better expected performance.

Bagging, achieving diversity by bootstrap aggregating the training data, improves the predication accuracy and also helps to reduce variance and alleviates the overfitting problem. Bagging was first implemented in CART(Classification And Regression Tree) [39] and usually applied with unstable decision trees methods, however, it can work with any type of base learners [42–44]. One of bagging’s features is that it supports parallel computing, which is an important advantage in training large data.

A typical bagging algorithm [39] is presented in the following Table 1:

**Table 1:** Algorithm 1. Bagging.

1: Let *T* be the set of *n* training examples (**x** ^*i*^, *y*_*i*_), *i* ∈ 1, 2, ⋯, *n*.
2: *B* is the number of base learners and *L* the base learning algorithm.
3: for(*i* = 0; *i* < *B*; *i* + +){
4: Create a bootstrapped training set *T*_*i*_ of size *n* by sampling with replacement.
5: Learn a specific base learner *L*_*i*_(x, *y*) on *T*_*i*_ by using *L*.
6: }
7: The final learning algorithm *C* is the ensemble of all base learners {*L*_*i*_} and a test example x* is classified by using a simple majority voting method: $$\begin{array}{c} y^{*}=\arg\;\max_{y}\sum_{L_{i}\in C}L_{i}\left(x^{*},y\right) \end{array}$$

In each bootstrap training set, about 37% of the original data examples will be left out(also called Out Of Bag or OOB data) and this can be used as the validation dataset. It is noticed that bagging is particularly useful when there is a limited data [20].

## The Proposed Lasso Ensemble Algorithm

In this section, we first present the idea on how to balance the data and then we describe how to generate and aggregate the diversified base learners within the ensemble. Later on, we provide two kinds of measures to assess variable importance. Finally, we describe the proposed algorithm.

### Balancing the data

In real credit scoring applications, most would-be-default applicants have either given up the applications or been rejected immediately after a first screening, thus in most collected data, only a small portion of the applications are default(minority) ones. However, as we do not have a good understanding of who would be defaulted in the future, we need a lot of non-default(majority) examples to help the classification. Usually, the non-default examples are collected to complement the default ones and thus these non-default examples are chosen for various reasons and may be from different sub-populations.

**Fig 1:**
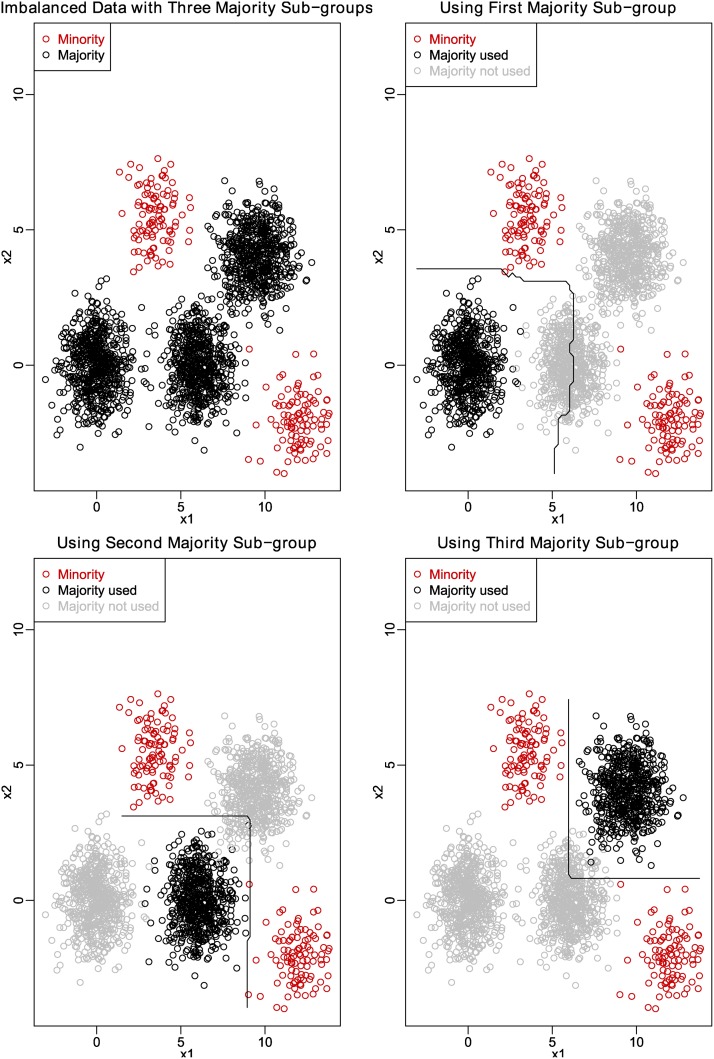
Classification when majority are from different populations.

As shown in the upper left panel of Fig. 1, majority data greatly outnumber minority data in the training set and it is non-trivial to find a satisfactory classification model. However, when majority examples are or seem to be from different populations (in this examples, majority data are generated from three different distributions), it is easy to train a sub-model or base learner with high classification accuracy on each distribution of the majority data and all the minority data. From the upper right, lower-left, and lower-right panels of Fig. 1, we can see that when only one sub-group majority examples are considered, it is rather easy to find a satisfactory decision boundary between this sub-group of majority data and the whole minority data.

Thus, if we are given a large dataset, we can divide the available majority examples into a number (*k*) of subgroups according to some certain criterion (for example, variable similarity). Then, we can choose one subgroup of majority examples and all the minority examples to form a roughly balanced sub-training set. As a training set containing 1500–2000 examples of each class will be sufficient for building a near-optimal classifier in credit coring [29], the new sub-training set should be large enough for a classifier to maintain modest classification performance. Base classifiers built upon these balanced sub-training sets should have better performance on both majority and the minority class examples than classifiers trained with the original highly unbalanced dataset.

### Diversifying the data

In ensemble learning, the most popular method to achieve classifier diversity is using different training datasets to train individual base classifiers [37]. This is also the approach adopted in this study. Data diversity in this paper is realized by using different sub-groups of majority examples plus bagging versions of minority examples.

First, as mentioned above, majority data can be segmented into a number (*k*) of sub-groups using a clustering algorithm, and the examples within each sub-group are surely quite different from data in other sub-groups. As data size may vary from sub-group to subgroup, to eliminate those with a few examples, only subgroups have sizes larger than a threshold quantile (75%) are retained. Second, in the absence of adequate minority examples, resampling techniques such as bagging can be used for drawing overlapping random subsets of the available minority data. Thus, a sub-group of majority data and a bagging version of minority data can be combined together to train a different base classifier in creating a diversified ensemble. In this way, model performance degrade due to class imbalance is alleviated and diversity of classifiers in the ensemble is increased.

In the following, for ease of explanation, the currently used balanced sub-training set, and all the training data excluding the currently used sub-training set are called Balanced Sub-training set(BS), and Out of Balanced Sub-training set (OBS) data, respectively.

### Aggregating base learners

The most popular aggregation method in ensemble algorithms is majority voting which adopts class label predicted with the most votes. However, simple majority voting does not reflect the individual confidence of the base learners and the overall predicting power might be lowered [45]. It was also proved in [46] that in most cases, weighted average is better than simple majority voting. In the proposed method, when an ensemble of classifiers is constructed, unique voting weights to each classifier in the ensemble are assigned and the final prediction is judged on the weighted average of all base learners’ predictions. We also note that base classifiers with large weight values might dominate the final decision of the ensemble. Thus, to reduce the side effects of large weight values, we use the following sigmoid function as a scaling function in weight transformation:

$$\begin{array}{c} w_{i}=\frac{1}{1+e^{-p_{i}}} \end{array}$$ (4)

where *w*_*i*_ denotes the weight of the *i*-th base classifier, and *p*_*i*_ the performance of classifier *i* on the *i*-th validation data-*OBS*_*i*_.

### Evaluating variable importance

Determining which variables in a model are its most important predictors (in ranked order) is of vital importance in the interpretation of a model. In this study, we introduce two kinds of variable important measures, namely the LLR-occurrence measure and the mean AUC decrease measure.

The LLR-occurrence measure is based on variable selection property of Lasso logistic regression. Different variables are selected automatically in building each LLR base learner in LLRE. The presence or absence of a predictor variable in the Lasso model naturally indicates whether it is closely related to the outcome variable or not. It is reasonable to evaluate the importance of different variables by ranking the variable occurrences among all iterations. A larger LLR-occurrence value will imply a more important variable and vice versa. Note that the largest possible LLR-occurrence value is the ensemble size. The variable importance for variable *j* in terms of LLR-occurrence is formally defined by:

$$\begin{array}{c} VI_{j}^{occu}=\sum_{i=1}^{L}I(\beta_{j}^{\left(i\right)}\neq 0) \end{array}$$ (5)

where *L* denotes the number of LLR model within the ensemble, *I*() the indication function, and $\beta_{j}^{\left(i\right)}$ the coefficient of variable *j* in the *i*−th LLR base model.

Many ensemble learning methods such as random forests can output variable importance based on error rate [40]. However, error rate (1- accuracy) always prefers the majority class and may not be appropriate for imbalanced class problems. As AUC measure is independent of class distribution and can be used to measure variable importance when facing imbalanced classes. Thus, in this study, we introduce a new importance measure, mean decrease AUC value in base classifiers to determine variable importance. It is based on the difference between the OBS data AUC values before and after permuting the values of the variables in question. The variable importance for variable *j* in terms of mean AUC decrease is defined by:

$$\begin{array}{c} VI_{j}^{auc}=\frac{100}{L}\sum_{i=1}^{L}\left(AUC_{j}-AUC_{\bar{j}}\right) \end{array}$$ (6)

where *AUC*_*j*_ and $AUC_{\bar{j}}$ denotes AUC value on the current OBS data before and after the permutation of variable *j*. If the variable in question is not associated with the outcome, permutating its values will have no influence on the classification and hence no influence on the AUC. On the contrary, if outcome and variables are indeed associated, dropping this variable by permuting its values results in a worse classification and will lead to a decrease in the AUC value. The difference in AUC before and after randomly permuting the variable will reflect the importance level of this variable. Averaging the decrease across all the LLR base models, we will get a list of mean decrease AUC values. The higher a mean decrease AUC value is, the more important a variable will be.

### The algorithm

In this study, Lasso(L1-norm penalty) is chosen as the base learner in that it is very efficient in dealing large-scale datasets and has a built-in variable selection property. However, computing the Lasso solution is a quadratic programming problem and non-trivial. In the latest related research [33, 42], the entire path of solutions with varied *λ* are computed using coordinate descent methods and the best *λ* is determined through cross-validation. Indeed, given a large dataset, we can also make use of the OBS data in each iteration as validation data to determine which value of *λ* is the best. The proposed Lasso-logistic regression ensemble(LLRE) algorithm is presented in Table 2:

**Table 2:** Algorithm 2. A Lasso-Logistic Regression Ensemble(LLRE) Algorithm.

1: INPUT:
2: *D* ← training data; *k* ← number of majority sub-groups
3: OUTPUT:
4: Ensemble of Classifiers *C*
5: **procedure** LLRENS(*D*, *k*)
6: Cluster the majority examples into *k* sub-groups using a clustering algorithm such as K-means and discard sub-groups whose size are less than the upper quantile (75%)
7: The ensemble size *L* ← 0.75 * *k*
8: **while** *i* ≤ *L* **do**
9: Select the *i*−th sub-group majority data *Maj*_*i*_
10: Generate a bootstrap sample of the minority data *Min*_*i*_
11: Train a LLR model *C*_*i*_ on data *BS*_*i*_ = *Maj*_*i*_ + *Min*_*i*_
12: Evaluate *C*_*i*_ with all possible *λ*s on *OBS*_*i*_ = *D* − *BS*_*i*_ and choose *λ* with the best performance
13: Record the performance *p*_*i*_ of *C*_*i*_ with the best *λ* on *OBS*_*i*_
14: Calculate *C*_*i*_’s weight *w*_*i*_ according to formula (4)
15: **end while**
16: **return** the ensemble *C*
17: **end procedure**
18: In prediction, a sample (*x, y*) is assigned with class label y* according to: $$\begin{array}{c} y^{*}=\arg\;\max_{y}\sum_{C_{i}\in C}w_{i}*C_{i}\left(x,y\right) \end{array}$$

From Table 2, we know that similar to bagging, LLRE is also a parallel algorithm and the “while” part of the algorithm can be executed concurrently. When facing large data, LLRE can make full use of the available parallel computing facilities to save training time.

## Experiment Results

In this section, we first describe the data set used in the experiments and then the performance metrics and statistical tests that we used. Finally, we present and discuss the experimental results.

### Dataset

In this study, we want to test our LLRE algorithm’s performance on large real credit scoring data. However, the only available large credit scoring dataset is from a data mining competition, publicly available on Kaggle.com. In the data, a bad customer is defined “default” (class 1) as some one would experience financial distress in the next two years as of the approval date. The size of the credit data used is 150,000 and the original variables, i.e. variables available in the above Kaggle dataset include age, debt ratio, income, number of dependents and six other personal financial variables. The vast majority (139,974) of the dataset is from class 0 and the minority (10,026) is from class 1. It is obvious that the ratio (about 14:1) between the two classes is obviously unbalanced.

### Performance metrics and statistical tests

In this study, we use two measures commonly used in the unbalanced classification literature to measure the performance of all algorithms. Accuracy is inappropriate here in that when high class imbalance presents in the data, even a simple solution of classifying all default(minority) cases into the majority class will show a high and satisfactory performance value. One measure chosen in the experiments is the area under the ROC (Receiver Operating Characteristics) curve (AUC) as suggested by [9, 34] and the other measure is F-measure(the harmonic mean of precision and recall) which is also frequently used in unbalanced classification and credit scoring [47, 48].

In order to compare the performance values of different classifiers, the non-parametric Friedman test [49], which is based on the average ranked performance of the classification algorithms on each run of the dataset, is adopted. Friedman’s test statistic is calculated as follows:

$$\begin{array}{c} FT=\frac{12}{nm(m+1)}\sum_{j=1}^{m}{\left(\sum_{i=1}^{n}r_{i}^{j}\right)}^{2}-3n\left(m+1\right) \end{array}$$ (7)

where *n* denotes the number of runs, *m* the number of classifiers, and $r_{i}^{j}$ the rank of classifier *j* on the *i*-th run. For a large sample *n* (i.e. greater than 5), the statistic follows a Chi-square distribution with *m*−1 degrees of freedom: *FT* ∼ *χ* ^2^(*m* − 1). If the value of *FT* is large enough, we can reject the null hypothesis that there is no significant difference among the different classifiers and a post-hoc test such as the Nemenyi test [49] can further be applied to pinpoint where the difference lies.

In the Nemenyi test, denote by *R*_*j*_ the mean rank of classifier *C*_*j*_ on all runs of the dataset: $R_{j}=\frac{1}{n}\sum_{i=1}^{n}r_{i}^{j}$. Then for any two classifiers *C*_1_ and *C*_2_, the statistic *z* is calculated as follows:

$$\begin{array}{c} z=\frac{R_{j1}-R_{j2}}{\sqrt{\frac{m(m+1)}{6n}}} \end{array}$$ (8)

The performance of two classifiers is significantly different if the *z* value is larger than the critical difference based on the Studentized range statistic divided by $\sqrt{2}$ [49].

### Results and discussions

We conduct our experiments on a system with a Pentium Dual-Core 3.20GHz CPU and 4G RAM. The proposed LLRE algorithm is implemented in the R programming language. In the experiments, training/testing sets are from a random 80% and 20% split of the Kaggle dataset.

#### Variables generation

In the Kaggle dataset, beside the outcome variable “SeriousDlqin2yrs”, there are 10 predictor variables namely “Revolving utilization of unsecured Lines”, “Age”, “Number of time 30–59 days past due not worse”, “Debt ratio”, “Monthly income”, “Number of open credit lines and loans”, “Number of times 90 days late”, “Number of real estate loans or lines”, “Number of times 60–89 days past due not worse” and “Number of dependents”. In order to create a diversified variable set for logistic regression base models, we need to generate more variables or do some variable transformation based on these original variables.

As suggested in [50], in the case of high-skewed data distribution, a log transformation for numeric variables is necessary as the logarithm function can squeeze together the larger values and stretch out the smaller values and hence make the patterns in the data more visible and interpretable. If the predictors variables contain some zeros, we may use a log transformation variant–*log*(*x*+1) where *x* is the predictor value to ensure a similar effect [51]. This is the major technique applied in our variable generation process.

After careful study of the Kaggle data, we find some special code values(a common practice in survey data) exist for predictor variables such as “Number of time 30–59 days past due not worse”. These codes do not denote the expected “Number of time” but really mean something else not mentioned by the data provider. We also notice that there are quite a few missing(NA) values in the Kaggle data and these NA values generally denote some certain financial information [32]. Consequently, replacing these NA values by a mean or median imputation could degrade classifiers’ performance. It is better to keep these unusual values (include NA observations) by adding some new variables [52]. This is another technique used in obtaining new variables.

The third and last major variable generation technique applied in this study is binning or discretization, which reduces the cardinality of continuous and discrete data by grouping related values together into a small number of bins [53]. For example, variables as “Age” and “Monthly income” can be further divided into several intervals to reduce the noise or non-linearity and hence improve the model’s predicative power. In our study, usually two or more generation techniques are combined together for a single variable. Take the original “Age” variable as the example, the minimum value is “0” and the maximum value is “109” and the mean value is 52.3. Three new variables based on “Age” can be generated according to the following formula:

$$\begin{array}{c} GeneratedAgeVariables\left\{\begin{array}{cc} Juvenile\_Age, & 1\;,if\;\;age\leq 17\\ Working\_Age\_log, & log(age-17),\;if\;\;59\leq age\leq 18\\ Senior\_Age, & 1\;,if\;\;age\geq 60 \end{array}\right. \end{array}$$ (9)

All together 80 derived variables are generated from the original variables and thus the derived data set contains 150,000 examples with 80 generated variables.

We run LLRE, LLR, RF and CART algorithms on the Kaggle dataset using the above two different set of variables and the experiment results in terms of AUC are shown in Table 3.

**Table 3:** Performance on two variable sets in terms of AUC.

Classifier	Original variables	Generated variables
LLRE	0.6796	0.8597
RF	0.8488	0.8587
LLR	0.4898	0.8567
CART	0.7702	0.7632

From Table 3, we can see that performance of most classifiers is improved and LLRE and LLR algorithms even get an increase of about 20% or more in AUC. This demonstrates the usefulness of the generated variables. We also notice that performance of RF (a recursive partitioning ensemble) does not improve too much on the generated variables, as a large part of our variable generation is based on partitioning the original variables into specific intervals.

#### Choice of *k*

**Fig 2:**
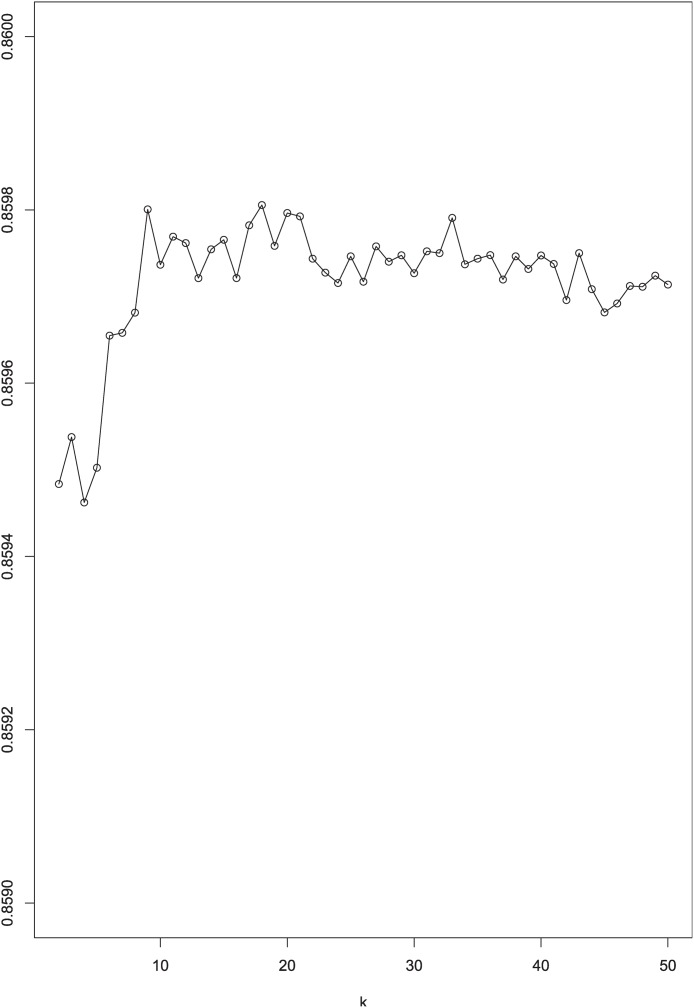
AUC change with LLRE using different *k*.

First, we want to test the performance of LLRE with different values of *k*. As shown in Fig. 2, LLRE’s AUC values increase slowly when the number of iterations increases at the very beginning. This indicates that clustering the majority ensemble does lead to a better performance. And a larger ensemble size also implies an increase in AUC but when *k* ≥ 10, LLRE is no longer too much sensitive to the parameter *k*.

From our empirical study, a suggested value for *k* can be given by the function *k* ≥ *max*(*ceil*(*N*2/*N*1), 10) to ensure data balance and classification accuracy, where *N*_1_ and *N*_2_ denote the number of minority examples and number of majority examples in the data. Hence, the sub-group number parameter *k* for LLRE in the following experiments is set to 14.

#### Variables importance

**Fig 3:**
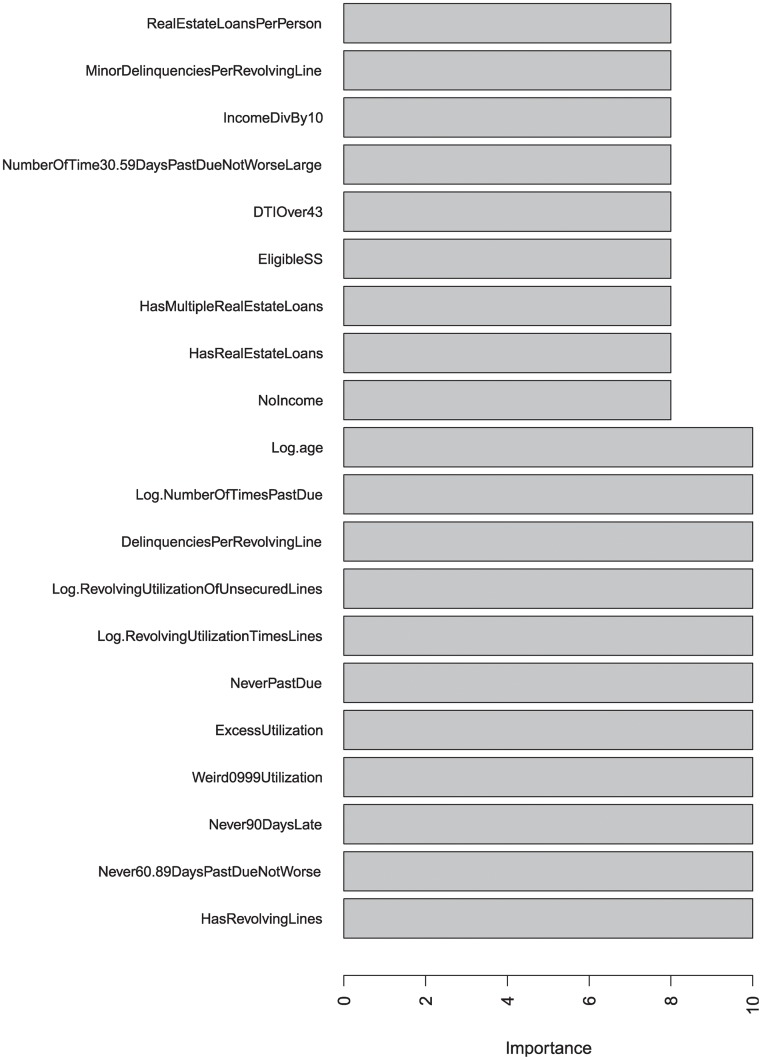
Top 20 important variables in terms of LLR occurrence.

**Fig 4:**
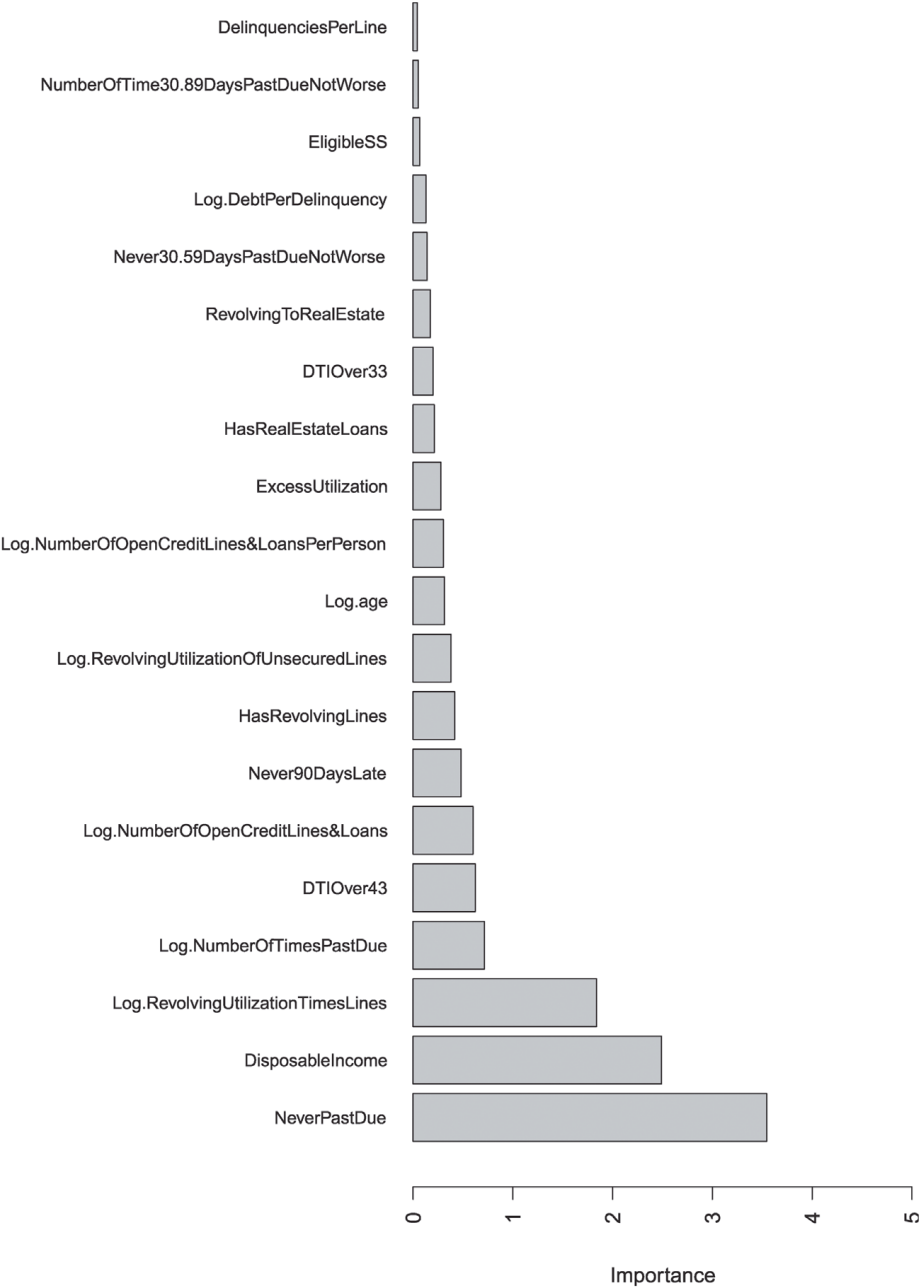
Top 20 important variables in terms of mean AUC decrease.

We use the LLR-occurrence measure and the mean AUC decrease measure defined in the previous section to measure variable importance in the experiment. Bar plots of top 20 important variables in terms of LLR-occurrence and mean AUC decrease are shown in Figs. 3 and 4, respectively.

From Fig. 3, variables including “Has Revolving Lines”, “Never 60–89 Days Past Due Not Worse”, “Never 90 Days Late”, “Delinquencies Per Revolving Line”, “Weird0999 Utilization”, “Excess Utilization”, “Never Past Due”, “Log Revolving Utilization Times Lines”, “Log Revolving Utilization Of Unsecured Lines”, “Log Number Of Times Past Due” and “Log Age” are included in all LLR base models. These variables are the most important ones in terms of LLR-occurrence measure.

From Fig. 4, we can see that “Never Past Due”, “Disposable Income” (based on original variables “Debt Ratio” and “Monthly Income”) and “Log Revolving Utilization Times Lines” are very important indicators of whether the applicant will default in the future, as permutation of these two variables can cause a decrease in AUC of more than 1 percent on average.

As is shown in Figs. 3 and 4, the two measures agree strongly on most important variables. Different measures give similar results provide further confidence that these variables can be used in credit scoring to evaluate the default risks of credit applicants.

According to the experiment results, variables such as number of dependents or household size provide the least information for evaluating an applicant’s future credit rating.

#### Comparison study

Here, we want to compare our approach with other popular credit scoring approaches. The winning algorithm for the Kaggle competition is a blended approach of multiple random forests, support vector machines data cleaning, and gradient boosting machines [54]. As the algorithm is not public available and is only slighted better (0.005) than random forests in terms of AUC [54], hence random forests(RF) can be regarded as the start-of-the-art credit scoring algorithm for the Kaggle data. Moreover, as the time complexity of standard SVM algorithm and MLP neural networks are usually greater than *O*(*n* ^2^), a single run of these algorithms on desktop machines will take even a month or more to complete and this makes them inappropriate for large scale classifications.

Thus, in this study, we only compare LLRE with popular single and ensemble learning techniques with fast training time such as Lasso-Logistic Regression (LLR), CART decision tree and RF. Comparison with these models are conducted with corresponding “glmnet”, “randomForest”, “rpart” packages in R. In the experiments, we want all classifiers to have the same opportunities to achieve the best results, thus the default settings are adopted: for LLR, the error distribution and link function *family* = *binomial*, type.measure = *auc*, *alpha* = 1; for CART, the split criteria is set to Gini; for RF, number of trees *ntree* = 500 and split variables *mtry* = 3. The following Tables 4 and 5 report the AUCs and F-measure values of the proposed LLRE, RF, LLR and CART algorithms on 30 runs of the experiments.

**Table 4:** Performance comparison in terms of AUC.

Run No	LLRE	RF	LLR	CART
1	0.8598	0.857	0.8571	0.7632
2	0.8553	0.8538	0.8526	0.7676
3	0.8662	0.8609	0.8651	0.7786
4	0.8602	0.8576	0.8577	0.7778
5	0.858	0.8564	0.8559	0.7746
6	0.8662	0.8628	0.8638	0.7689
7	0.8544	0.8536	0.8526	0.77
8	0.8619	0.8617	0.8589	0.7749
9	0.8657	0.8606	0.8636	0.7832
10	0.8575	0.8569	0.8561	0.7665
11	0.8622	0.8578	0.8604	0.7762
12	0.8565	0.8551	0.8542	0.7748
13	0.8576	0.8519	0.8573	0.7763
14	0.8573	0.8537	0.8547	0.7761
15	0.8638	0.8648	0.8606	0.7699
16	0.8567	0.8535	0.8547	0.7728
17	0.8586	0.8579	0.8558	0.7783
18	0.8696	0.8631	0.8666	0.7792
19	0.8529	0.8523	0.8506	0.77
20	0.8651	0.8607	0.8609	0.7732
21	0.8537	0.8498	0.8506	0.7695
22	0.8652	0.8625	0.8635	0.7783
23	0.8603	0.8606	0.8578	0.7738
24	0.8607	0.856	0.8584	0.7692
25	0.8668	0.8642	0.8648	0.7763
26	0.859	0.8562	0.857	0.7698
27	0.8574	0.8553	0.8548	0.7807
28	0.8576	0.8543	0.8564	0.7685
29	0.8633	0.8599	0.8609	0.7685
30	0.8636	0.8617	0.8623	0.7775

**Table 5:** Performance comparison in terms of F-measure.

Run No	LLRE	RF	LLR	CART
1	0.3856	0.2683	0.2599	0
2	0.3872	0.2727	0.2466	0
3	0.3777	0.2712	0.2626	0
4	0.3799	0.2694	0.2738	0
5	0.4004	0.2873	0.2793	0
6	0.3848	0.2699	0.2548	0
7	0.3756	0.2671	0.2555	0
8	0.394	0.2834	0.2575	0
9	0.4088	0.2933	0.2796	0
10	0.3729	0.2734	0.265	0
11	0.3759	0.2669	0.2647	0
12	0.3846	0.2725	0.2567	0
13	0.3939	0.2869	0.2759	0
14	0.3717	0.2815	0.2615	0
15	0.3806	0.2689	0.2672	0
16	0.3855	0.2877	0.2785	0
17	0.3963	0.2756	0.2677	0
18	0.372	0.2679	0.2466	0
19	0.3751	0.2579	0.2642	0
20	0.3807	0.2817	0.2664	0
21	0.3737	0.2804	0.2559	0
22	0.3836	0.2648	0.2604	0
23	0.3937	0.2699	0.2627	0
24	0.3876	0.2811	0.278	0
25	0.3951	0.2958	0.2823	0
26	0.3784	0.2744	0.2548	0
27	0.3895	0.2779	0.2648	0
28	0.3815	0.2621	0.2413	0
29	0.3716	0.2723	0.2517	0
30	0.3826	0.2705	0.2649	0

The Friedman rank sum test statistic for the above AUC results is 77.88. This is significant (the corresponding p-value is less than 2.2e-16) and a post hoc Nemenyi test was then applied to find out which pairs of algorithms are significantly different.

For the ease of notation, classifiers LLRE, RF, LLR and CART are denoted by A, B, C, D respectively. The average ranks of classifiers A, B, C and D are:

$$\begin{array}{c} R_{jA}=1.0667,R_{jB}=2.5667,R_{jC}=2.3667,R_{jD}=4 \end{array}$$

Using the proposed algorithm (A) as the control and computing the *z* statistic of Nemenyi test for different classifier pairs, we obtain:

$$\begin{array}{c} z_{BA}=4.5,z_{CA}=3.9,z_{DA}=8.8 \end{array}$$

For *α* = 0.05, the critical value of Studentized range distribution *q*_*α*_ = 3.71 and divide *q*_*α*_ value by $\sqrt{2}$, we get the Nemenyi’s test critical value of 2.619. It can be seen that all three Nemenyi statistics $z_{BA}$, $z_{CA}$ and $z_{DA}$ values exceed 2.619 and thus there exists significant differences between the proposed LLRE and the other three algorithms. In other words, in terms of AUC, LLRE gives the best performance on the Kaggle dataset.

From Table 5, we can see that on the F-measure, LLRE shows remarkably the best performance, followed by RF, LLR and then CART. Similarly, the Friedman rank sum test statistic for the above F-measure results is 87.76. This is also significant (the corresponding p-value is less than 2.2e-16) and a post hoc Nemenyi test was then applied and we obtain the corresponding Nemenyi statistics:

$$\begin{array}{c} z_{BA}^{\prime }=3.2,z_{CA}^{\prime }=5.8,z_{DA}^{\prime }=9 \end{array}$$

Again, it can be seen that all three Nemenyi statistics $z_{BA}^{\prime }$, $z_{CA}^{\prime }$ and $z_{DA}^{\prime }$ values exceed 2.619. Thus, there also exists significant differences between the proposed LLRE and RF, LLR, CART when F-measure is used as an evaluation metric.

#### Discussions

Non-parametric models such as random forests have gained popularity in recent years with remarkable performance in credit scoring problems and it is also one of the most advanced statistical learning methods so far. Logistic regression has over the years a standard technique in credit scoring problems due to its well-understood feature and wide availability. However, its regularized version-lasso logistic regression for credit scoring problems is still limited. In this study, we examined the performance of the proposed Lasso-logistic regression ensemble, random forests, lasso-logistic regression, and classification and regression tree, for a large data credit scoring problem. Encouragingly, except classification and regression tree, all approaches have shown adequate ability in predicting default credit loans. Experiments results have shown that the combination of clustering and bagging techniques not only takes advantages of the superior capability of ensemble learning and but also provides an approach for extending the use of the traditional parametric models in credit scoring.

In the experiments, LLRE has demonstrated an overall better performance across the popular AUC and F-measure for unbalanced credit scoring. On the AUC measure, LLRE outperforms RF, LLR and CART significantly. The difference between LLRE and RF in terms of AUC appears marginal yet in fact constant. These marginal improvements should make an adequate gain in profits for financial institutions. On the F-measure, LLRE has shown a surprising well performance and surpassed all the other compared methods by a large margin. LLRE even beats RF by more than 10 percent in term of F-measure on average. We also notice that although CART might obtain a high performance in term of accuracy but its bad performance on the minority (default) cases make it the worst choice in unbalanced learning with zero F-measure values during all runs of the experiments.

Another factor contributing to the success of LLRE is possibly the carefully generated variables. Its better performance with the generated variable set implies that time-consuming exploratory data analysis and variable selection process using domain’s expertise is very important in constructing logistic regression like models. This finding agrees with the assertion made in [47]. When such preprocessing efforts are unavailable, random forests could be an alternative.

Our variable importance results are in line with previous studies that credit history related variables such as past credit due information and revolving utilization data are very important factors in predicting loan applicants’ quality [55–57]. As revealed by the mean AUC decrease measure, disposable income is another crucial factor in evaluating a customer’s repayment quality. This finding also agrees with the results reported previously [58, 59]. The “Log age” variable which appears both in Figs. 3 and 4 is also crucial in extending credit and this finding agrees with [56, 59] but is inconsistent with a previous research [55] suggesting that age was one of the least differentiating variables. In our study, the least important factor selected by both importance measures is the number of dependents of the applicant. However, this finding does not agree with the results in [60] and needs further investigation.

## Conclusions

In this paper, a new ensemble credit scoring algorithm based on Lasso-logistic regression for large unbalanced data has been presented. In our approach, clustering the majority data and bagging the minority data have been applied to generate balanced training data and maintain data diversity. Different from previous researches, the *λ* parameter is determined by OBS data, which also helps to reduce training time of base LLR models. Experiment studies using real world credit scoring data have demonstrated that LLRE beats CART, LLR and RF in terms of AUC and F-measure.

We also show that the proposed LLRE method can be used to evaluating variable importance by providing two kinds of importance measures. As to which variables may influence the credit decision process, experiment results with the proposed algorithm using the LLR-occurrence and AUC mean decrease measures have shown that past due information, disposable income and revolving utilization times lines are the most important indicators.

Future researches may focus on newly developed fast and accurate clustering methodologies to produce better sub-groups of majority data. Choosing other state-of-the-art models with fast training time as the base classifiers could also be considered.
